# Cognitive behavior therapy for dizziness: A protocol for systematic review and meta-analysis

**DOI:** 10.1097/MD.0000000000022945

**Published:** 2020-12-24

**Authors:** Lin Li, Xiaoping Gao, Jianguo Liu, Xiaokun Qi

**Affiliations:** a The Second School of Clinical Medicine, Southern Medical University, Guangzhou; b Department of Neurology, Hunan Provincial People's Hospital, Changsha; c Department of Neurology, The Sixth Medical Center of PLA General Hospital, Beijing, P.R. China.

**Keywords:** cognitive behavior therapy, dizziness, meta-analysis, randomized controlled trials

## Abstract

**Objectives:**

To investigate the association between cognitive behavior therapy (CBT) and dizziness.

**Methods:**

The databases including PubMed, Embase, Cochrane Library and Web of science will be searched for randomized controlled trials. Weighted mean difference and 95% confidence interval will be utilized to calculate the effect of CBT on dizziness between the 2 groups.

**Conclusions:**

This meta-analysis will provide a high-quality synthesis from existing evidence for the relationship between CBT and dizziness.

**OSF registration number:**

10.17605/OSF.IO/YNH5W

## 1 Introduction

Dizziness is a common functional disorder of the brain, which is characterized by fullness in head, top-heavy, shaking in the brain and giddiness. Approximately 20% to 30% of general population experience dizziness. Its incidence increases with age ^[1–3]^ and rises about 10% per 5 years. Previous studies reported that dizziness is associated with balance, vestibular, cardiovascular and psychological disorders.^[4–7]^ A sizeable proportion of patients who visit in neurology clinic suffer from dizziness caused by psychologic factors, most observably anxiety.^[8–10]^ Patients with dizziness may fear the falling, limited daily activities and other illnesses.

At present, several methods have been applied for the treatment of dizziness, such as drug therapies, vestibular rehabilitation (VR) and cognitive behavior therapy (CBT). Of which, the drug therapies focus on treating patients with active vestibular disease. Although these medications can relieve dizziness to some extent, not all patients benefit.^[11–13]^ Studies on VR have also been found to improve clinical outcomes in patients with dizziness.^[14–17]^ It promotes central nervous system compensation for vestibular defects through specific movements. While the effectiveness of VR in patients without vestibular deficits who manifested psychological symptoms is limited. Early researches showed that CBT is effective for the early treatment of dizziness, which is centered on behavior in patients’ own environment and graded exposure. CBT may reduce anxiety and depression associated with persistent dizziness, and also avoid certain activities such as related head movements.

Given the development of CBT in health psychology and medicine, as well as its effectiveness in treating various physical ailments, such as chronic pain. CBT may be equally important in the dizziness management. To the best of our knowledge, however, the efficacy of CBT in improving dizziness was inconsistent in the previously mentioned studies. Herein, we present a meta-analysis, aiming to assess the association between CBT and dizziness based on randomized controlled trials (RCTs).

## 2 Material and methods

The data in this study were accessed from openly available datasets, thus there was no need to get the approval from the Institutional Review Board of hospital.

### 2.1 Protocol registration

Prospective registration of this study has been approved by the Open Science Framework registries (https://osf.io/registries), and the registration number is 10.17605/OSF.IO/YNH5W. The protocol was written following the Preferred Reporting Items for Systematic Reviews and Meta- Analyses Protocols statement guidelines.^[18]^

### 2.2 Search strategy

This study will be performed according to the preferred reporting items for systematic reviews and meta-analyses. The systematic literatures will be searched for reported studies regarding CBT associated with dizziness. The databases including PubMed, Embase, Cochrane Library and Web of science will be updated on September, 2020. The searching strategies from PubMed are “Dizziness” OR “Dizzyness” OR “Orthostasis” OR “Lightheadedness” OR “Light-Headedness” OR “Light Headedness” AND “Cognitive Behavioral Therapy” OR “Behavioral Therapies, Cognitive” OR “Behavioral Therapy, Cognitive” OR “Cognitive Behavioral Therapies” OR “Therapies, Cognitive Behavioral” OR “Therapy, Cognitive Behavioral” OR “Therapy, Cognition” OR “Therapy, Cognitive Behavior” OR “Cognition Therapy” OR “Cognition Therapies” OR “Therapies, Cognition” OR “Cognitive PsychCBT vs wait-listapy” OR “Cognitive PsychCBT vs wait-listapies” OR “PsychCBT vs wait-listapies, Cognitive” OR “PsychCBT vs wait-listapy, Cognitive” OR “Therapy, Cognitive” OR “Cognitive Therapies” OR “Therapies, Cognitive” OR “Cognitive Therapy” OR “Cognitive Behavior Therapy” OR “Behavior Therapies, Cognitive” OR “Cognitive Behavior Therapies” OR “Therapies, Cognitive Behavior” OR “Behavior Therapy, Cognitive.”

### 2.3 Study selection

Inclusion criteria:

(1) RCTs;
(2) patients with dizziness;
(3) English articles;
(4) the CBT group: patients who received the CBT (including CBT combined with drugs or other therapies), and the control group: no treatments or merely drugs/other therapies.

Excluded criteria:

(1) conference abstracts, reported meetings, reviews or Meta-analyses;
(2) laboratory studies;
(3) researches without patients.

### 2.4 Methodological quality appraisal and data extraction

The modified Jadad scale will be used to identify high-quality researches. Methodological quality will be independently assessed by 2 authors (X Gao and J Liu). A third author (L Li) will validate, when the dissent is presented. According to the modified Jadad, the scores of included articles ≥4 will be defined as high-quality.

The data will be extracted from included studies containing authors, year of publication, the numbers of cases and controls, gender, mean age, quality of study, and score.

### 2.5 Assessment indicators

The efficacy of CBT in dizziness will be assessed on the basis of various scales. These scales will include Dizziness Handicap Inventory, Hospital Anxiety Scale, Hospital Depression Scale, Dizziness Symptoms Inventory, Safety Behaviours Inventory, Clinician-Administered PTSD Severity Scale, Orthostatic Panic Attack Severity Scale, Orthostatic Panic Flashback Severity Scale and Anxiety Sensitivity Index.

### 2.6 Statistical analysis

Meta-analysis will be analyzed using STATA 15.1 software (Stata Corporation, College Station, TX). Weighted mean difference and 95% confidence interval will be utilized to calculate the effect of CBT on dizziness between the 2 groups. *I*^*2*^ test will be used to access the heterogeneity of the scores. If *I*^*2*^≥50%, the random-effect model will be conducted. When *I*^*2*^ < 50%, the fixed-effect model will be used to analyze. Publication bias will be performed by Begg test. *P* < .05 will be considered statistically significant.

## 3 Discussion

The Methodology and quality of reporting in meta-analyses are crucial to public health and clinical decision-making. Although numerous studies have been reported, there is no consensus on the association between CBT and dizziness. In the current meta-analysis, we assessed the role of CBT in dizziness based on RCTs.

## Author contributions

LL and XKQ designed the study. LL, XPG, and JGL collected and analyzed the data. LL wrote the manuscript. XKQ critically reviewed, edited and approved the manuscript. All authors read and approved the final manuscript.

**Conceptualization:** Lin Li.

**Data curation:** Lin Li.

**Formal analysis:** Xiaoping Gao.

**Methodology:** Jianguo Liu.

**Project administration:** Xiaoping Gao, Jianguo Liu.

**Supervision:** Xiaokun Qi.

**Writing – review & editing:** Xiaokun Qi.
